# Differential gene and protein expression of chemokines and cytokines in synovial fluid of patients with arthritis

**DOI:** 10.1186/s13075-016-1196-6

**Published:** 2016-12-13

**Authors:** Anastasiya Muntyanu, Fatima Abji, Kun Liang, Remy A. Pollock, Vinod Chandran, Dafna D. Gladman

**Affiliations:** 1 Psoriatic Arthritis Program, Centre for Prognosis Studies in the Rheumatic Diseases, Krembil Research Institute, University of Toronto, University Health Network, 399 Bathurst Street 1E-410B, Toronto, ON M5T 2S8 Canada; 2 Department of Statistics and Actuarial Science, University of Waterloo, Waterloo, ON Canada; 3 Division of Rheumatology, Department of Medicine, University of Toronto, Toronto, ON Canada; 4 Krembil Research Institute, Toronto Western Hospital, Toronto, ON Canada; 5 Department of Laboratory Medicine and Pathobiology, University of Toronto, Toronto, ON Canada

**Keywords:** Biomarkers, Chemokines, Cytokines, Psoriatic arthritis, Synovial cells, Synovial fluid

## Abstract

**Background:**

Psoriatic arthritis (PsA), an inflammatory musculoskeletal disease, develops in approximately 30% of patients with psoriasis. Previously, chemokine (C-X-C motif) ligand 10 (CXCL10) was identified as a predictive biomarker of PsA in patients with psoriasis and was reduced after development of PsA. The purpose of the present study was to explore messenger RNA (mRNA) and protein expression of CXCL10 and its receptor, chemokine (C-X-C motif) receptor 3 (CXCR3), in the joints of patients with PsA to gain insight into their role in the pathogenesis of the disease.

**Methods:**

Sera from 47 patients with PsA and 33 healthy control subjects were compared for expression of CXCL10 by Luminex assay. Synovial fluid (SF) was obtained from patients with PsA (*n* = 40), osteoarthritis (OA; *n* = 14), gout (*n* = 8), and rheumatoid arthritis (RA; *n* = 11) during clinical care. SF mRNA and protein expression of *CXCL10*, interleukin-17A (*IL-17A*), *CXCR3*, *TBX21*, *RORC* and/or interferon γ (*IFNγ*) were compared among the above-mentioned disease groups, as well as in paired SF and serum samples from patients with PsA using real-time polymerase chain reaction and Luminex assays, respectively.

**Results:**

Serum CXCL10 was significantly higher in patients with PsA than in control subjects (*p* = 0.0007). *CXCL10*, *IL-17A*, and *TBX21* expression were elevated in SF cells of patients with PsA compared with those of patients with OA and gout, but not those of patients with RA. *CXCR3* and *RORC* were elevated in PsA SF cells compared with all other patient groups. Concordant results were obtained for CXCL10 and IL-17A protein expression. IFNγ was elevated in PsA SF compared with OA SF (*p* = 0.015). CXCL10 protein expression was substantially increased in SF (median 7283.9 pg/ml, interquartile range [IQR] 1330–10,362 pg/ml) compared with paired serum samples (median 282.06, IQR 180.7–395.8 pg/ml; *p* = 0.001), whereas IFNγ was significantly reduced (SF median 6.03 pg/ml, IQR 4.47–8.94 pg/ml; versus serum median 23.70 pg/ml, IQR 3.2–104.6 pg/ml; *p* = 0.001).

**Conclusions:**

CXCL10 may have an important etiological role in PsA that is analogous to that in RA, and it is a candidate biomarker to distinguish PsA from healthy individuals and from patients with OA and gout.

## Background

Psoriatic arthritis (PsA) is an immune-mediated inflammatory musculoskeletal disease that affects approximately 30% of patients with psoriasis [1–3]. PsA is associated with joint, ligament, and tendon pain, stiffness, and swelling that lead to damage to the peripheral, axial, and entheseal structures, resulting in reduced quality of life and life expectancy for the affected individuals [4, 5]. For individuals affected by PsA, the initial screening and diagnosis are often done by family physicians or dermatologists. Studies have shown that approximately 50% of cases of PsA are currently undetected in patients with psoriasis [6, 7]. Physicians with limited experience in this area may find it difficult to diagnose PsA, which can be confused with other rheumatologic conditions such as osteoarthritis (OA) or gout. The identification of biomarkers that distinguish PsA from the general population and/or other rheumatologic diseases can aid in the development of tools for family physicians and dermatologists to better identify patients with PsA.

We previously performed microarray analysis to identify transcriptomic biomarkers that distinguish patients with PsA from those with psoriasis without PsA (PsC). We found that chemokine (C-X-C motif) ligand 10 (*CXCL10*) was upregulated in patients with PsA compared with those with PsC [8]. CXCL10 is a ligand for the chemokine (C-X-C motif) receptor 3 (CXCR3), and their interaction is responsible for recruitment of activated T helper (Th) cells and natural killer (NK) cells to the site of inflammation [9, 10]. In acute and chronic inflammation, leukocyte infiltration is regulated by both exogenous and endogenous factors, including cytokines, chemokines, and proteases [9]. It is generally accepted that the main endogenous inducer of CXCL10 is interferon γ (IFNγ); however, acting in conjunction with tumor necrosis factor α (TNFα), a synergic effect has been reported in several cell types, including leukocytes, epithelial cells, endothelial cells, and fibroblasts [11, 12]. Secreted CXCL10 proteins then recruit Th1 lymphocytes expressing CXCR3 to the sites of inflammation [11–13]. Together, this forms a positive feedback loop of inflammation and cell proliferation.

We have also previously shown that serum levels of CXCL10 were elevated in patients with psoriasis who subsequently developed PsA compared with those who did not develop PsA, and this was independent of clinical predictors of PsA [14]. Thus, CXCL10 may be important in the pathogenesis of PsA and may be a useful predictor of PsA in patients with psoriasis. CXCL10 levels in serum also dropped after the development of PsA in these patients, possibly as a result of an accumulation of activated lymphocytes in target tissues [14].

The objective of the present study was to further explore CXCL10 expression in serum and synovial fluid (SF) of patients with PsA. First, we compared serum CXCL10 expression in patients with PsA and in healthy control subjects, as well as SF CXCL10 expression in patients with PsA, OA, rheumatoid arthritis (RA), and gout. Next, we compared paired synovial and serum expression of CXCL10. In addition, the expression of CXCR3, IFNγ, and interleukin (IL)-17A, as well as the Th1- and Th17-specific transcription factors T-bet and RAR-related orphan receptor γt (RORγt), was measured to gain insight into the interplay of these molecules with CXCL10 in disease pathogenesis. The aim of this study was thus to support the use of CXCL10 as a biomarker of PsA susceptibility.

## Methods

### Study subjects

Patients with PsA with available SF samples from the knee joint were identified from a cohort of patients followed prospectively from 2004 to 2015 at the University of Toronto PsA clinic. The diagnosis of PsA was made by a rheumatologist, and the patients satisfied the ClASsification for Psoriatic ARthritis (CASPAR) criteria [15]. At baseline and at each 6- to 12-month follow-up visit, a complete history including demographic and disease-related features, as well as a physical examination including skin and musculoskeletal assessments, was carried out according to a standard protocol.

**Fig. 1:**
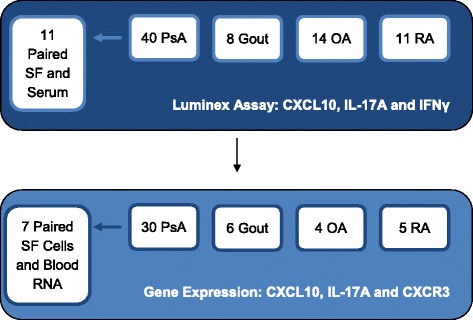
Summary of synovial fluid (SF) samples measured by Luminex assay (protein) and gene expression (messenger RNA). Gene expression was measured in a subset of patients analyzed with the Luminex assay. *CXCL10* Chemokine (C-X-C motif) ligand 10, *CXCR3* Chemokine (C-X-C motif) receptor 3, *IFNγ* Interferon γ, *IL* Interleukin, *OA* Osteoarthritis, *PsA* Psoriatic arthritis, *RA* Rheumatoid arthritis

SF from patients with PsA, gout, and RA was drawn from knee joints during clinical care. Samples from patients with OA were obtained during either routine joint aspiration or full knee replacement surgery. SF samples were centrifuged and stored in the biobank at −80 °C for Luminex assays (Luminex, Austin, TX, USA). When cells were present in SF, cell pellets were further processed for messenger RNA (mRNA) analysis, as described below. Samples obtained from SF for Luminex assay and gene expression experiments are summarized in Fig. 1. Additionally, serum samples were obtained from 47 patients with PsA and 33 healthy volunteers. This study was approved by the University Health Network Research Ethics Board according to the principles of the Declaration of Helsinki, and all participants provided written consent.

### Multiplex chemokine and cytokine assay

CXCL10, IL-17A, and IFNγ levels in SF were measured in patients with PsA (*n* = 40), gout (*n* = 8), OA (*n* = 14), and RA (*n* = 11) using a multiplex human chemokine/cytokine magnetic bead panel (EMD Millipore, Billerica, MA, USA) according to the manufacturer’s instructions. Serum samples from 11 patients with PsA, paired with SF drawn at the same visit, were also used to measure CXCL10, IL-17A, and IFNγ levels. Briefly, the SF or serum samples (25 μl) were incubated with a chemokine-/cytokine-specific capture antibody attached to magnetic beads. Then a detection antibody was added, followed by streptadavin-phycoerythrin acting as a reporter molecule. Data were acquired using the Luminex 200 system (Luminex, Austin, TX, USA ) and analyzed with Bio-Plex Manager software (Bio-Rad Laboratories, Hercules, CA, USA). All samples were measured in duplicate, and CXCL10, IL-17A, and IFNγ levels were quantified in relation to a fivefold serially diluted standard provided with the kit, using a five-parameter logistic regression curve.

### Gene expression analysis

Whole blood from seven patients with PsA was collected in Tempus® tubes (Life Technologies, Carlsbad, CA, USA), and mRNA was extracted according to the manufacturer’s instructions. SF cells were collected from 30 patients with PsA, 4 with OA, 6 with gout, and 5 with RA. The cells were treated with red blood cell lysis buffer and stored in TRIzol® reagent (Life Technologies). Phenol-chloroform extraction was used to extract total RNA, and the RNeasy® Mini Kit (Qiagen, Venlo, The Netherlands) was used to purify it. Reverse transcription of mRNA was performed using the Maxima First Strand cDNA Synthesis Kit (Life Technologies). *CXCL10*, *CXCR3*, *IL-17A*, T-bet (*TBX21*), and RORγt (*RORC*) complementary DNA was amplified with Platinum® SYBR Green qPCR SuperMix (Life Technologies) using corresponding forward and reverse primers. Reactions were measured in triplicate on a 7900HT real-time polymerase chain reaction system (Applied Biosystems, Foster City, CA, USA). C_t_ values obtained for *CXCL10*, *CXCR3*, *IL-17A*, *TBX21*, and *RORC* were normalized to *GAPDH* to generate ΔC_t_ values for each sample. Fold change between groups was determined by the ΔΔC_t_ method, where ΔΔC_t_ = mean ΔC_t_group1 − mean ΔC_t_group2 and fold change = 2^−ΔΔCt^. Values are expressed as 2^−ΔCt^ in the figures to show trends in gene expression [16].

### Statistical analysis

Gene expression differences of *CXCL10*, *CXCR3*, *IL-17A*, *TBX21*, and *RORC* between PsA and the other disease cohorts (OA, gout, and RA) were determined using Student’s *t* tests on ΔC_t_ values. For the comparison of chemokine/cytokine gene expression between SF cells and whole-blood RNA, paired Student’s *t* tests were performed. Differences between CXCL10, IL-17A, and IFNγ protein levels between groups were identified by performing the Wilcoxon signed-rank test for paired SF and serum samples and the Mann-Whitney *U* test for comparison between patients with PsA and control subjects. Statistical analysis was performed using the R statistical software package as well as Prism 5 software (GraphPad Software, La Jolla, CA, USA). For all the statistical tests performed, *p* < 0.05 was accepted as significant.

## Results

### Patient characteristics

SF samples were obtained from a total of 40 patients with PsA, 14 with OA, 8 with gout, and 11 with RA. The demographic and clinical characteristics of patients with SF samples analyzed by the Luminex assay and the subset of patients with synovial cells also measured for gene expression are found in Table 1. Patients with PsA analyzed by Luminex assay, as well as the subset also included in the gene expression assay, were significantly younger than the entire cohort of patients with OA (*p* < 0.0001 by one-way analysis of variance). A significant difference in the proportion of male and female patients was found (*p* = 0.003 by Pearson’s chi-square test). There were no significant differences in patients who were included in gene expression analysis as compared with those who were measured only with the Luminex assay, including the use of systemic agents such as disease-modifying antirheumatic drugs and biologic agents. Additionally, there were no significant differences in mRNA expression or protein levels in PsA SF in response to different medication regimens. It should be noted that detailed clinical data on patients with RA, gout, and OA were not available for comparison. Additionally, 47 patients with PsA (mean age 46 years, 43% males) and 33 healthy control subjects (mean age 53 years, 42% males) were included for the comparison of CXCL10 levels in serum.

**Table 1:** Demographic and clinical characteristics of study subjects with synovial fluid analyzed by Luminex assay and gene expression

	PsA		OA		Gout		RA		*p* Value^a^
	Luminex (*n* = 40)	mRNA (*n* = 30)	Luminex (*n* = 14)	mRNA (*n* = 4)	Luminex (*n* = 8)	mRNA (*n* = 6)	Luminex (*n* = 11)	mRNA (*n* = 5)	
Male sex	26 (65%)	18 (60%)	3 (25%)	0 (0%)	7 (87.5%)	5 (83.3%)	2 (20%)	0 (0%)	0.0003
Age, years^b^	44.4 (12.9)	45.1 (13.3)	69.1 (11.4)	79.0 (1.4)	61.3 (22.0)	56.5 (23.8)	49.0 (17.5)	52.3 (17.8)	<0.0001
Duration of psoriasis^c^	14 (7.3–21.8)	18 (10–24)	–	–	–	–	–	–	0.584
Duration of PsA^c^	9 (3–15)	10 (3–15)	–	–	–	–	–	–	0.755
PASI^c^	1.8 (0.6–4.2)	1.8 (0.6–4.5)	–	–	–	–	–	–	0.959
Number of swollen joints^c,d^	2 (1–3)	1 (1–3)	–	–	–	–	–	–	0.834
Number of tender joints^c,d^	2 (0–5)	1 (0–3.8)	–	–	–	–	–	–	0.435
Current use of NSAIDs	18 (45%)	12 (40%)	–	–	–	–	–	–	0.676
Current use of DMARDs	17 (43%)	14 (47%)	–	–	–	–	–	–	0.728
Current use of biologics	10 (25%)	9 (30%)	–	–	–	–	–	–	0.642

*Abbreviations: DMARD* Disease-modifying antirheumatic drug, *mRNA* Messenger RNA, *NSAID* Nonsteroidal anti-inflammatory drug, *OA* Osteoarthritis, *PASI* Psoriasis Area Severity Index, *PsA* Psoriatic arthritis, *RA* Rheumatoid arthritis

^a^One-way analysis of variance or Mann-Whitney *U* test (continuous variables) or Pearson’s chi-square test (categorical variables)

^b^Mean (SD)

^c^Median (interquartile range)

^d^Tender joints were determined clinically in 68 joints, swollen joints in 66 (excluding hips)

### CXCL10 is elevated in PsA serum

**Fig. 2:**
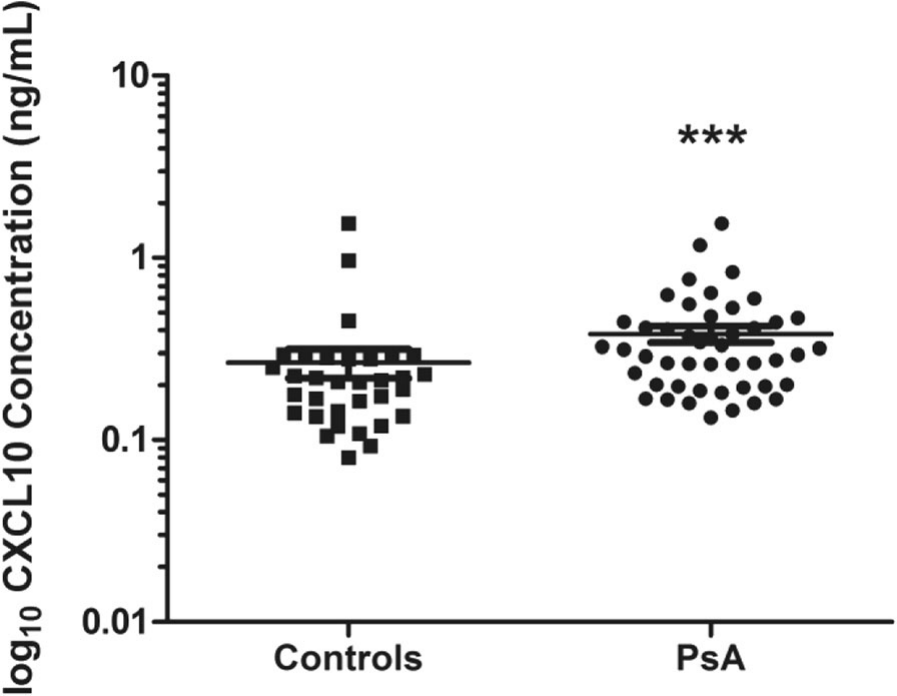
Scatter dot plot of serum chemokine (C-X-C motif) ligand 10 (CXCL10) expression from patients with psoriatic arthritis (PsA; *n* = 47) and healthy control subjects (*n* = 33). The data are presented as log_10_-transformed values to better visualize the absolute change in concentrations. CXCL10 levels were higher in patients with PsA (median 0.31 ng/ml, interquartile range [IQR] 0.20–0.44 ng/ml) than in healthy control subjects (median 0.21, IQR 0.14–0.28 ng/ml; *p* = 0.0007 by Mann-Whitney *U* test). ***p < 0.001

Our first step was to compare serum levels of CXCL10 between patients with PsA and healthy control subjects. As shown in Fig. 2, CXCL10 expression was significantly elevated in patients with PsA (median 0.31 ng/ml, interquartile range [IQR] 0.20–0.44 ng/ml) compared with control subjects (median 0.21, IQR 0.14–0.28 ng/ml, *p* = 0.0007). These results support the use of CXCL10 as a biomarker to distinguish PsA from the general population.

### CXCL10, IL-17A, and IFNγ protein levels are elevated in PsA synovial fluid

**Fig. 3:**
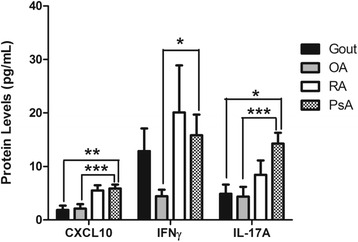
Bar graph of chemokine (C-X-C motif) ligand 10 (CXCL10), interferon γ (IFNγ), and interleukin (IL)-17A protein expression in synovial fluid (SF) from 40 patients with psoriatic arthritis (PsA), 14 with osteoarthritis (OA), 8 with gout, and 11 with rheumatoid arthritis (RA). Error bars indicate SEM. Asterisks are used to indicate significant differences between groups, where *p < 0.05, **p < 0.01, and ***p < 0.001 (Mann-Whitney *U* test). CXCL10 concentration was converted to nanograms per milliliter

Next, cytokine and chemokine protein expression was measured in SF from 40 patients with PsA, 14 with OA, 8 with gout, and 11 with RA. As shown in Fig. 3, CXCL10 levels (median 5.39 ng/ml, IQR 1.81–9.82 ng/ml) were significantly elevated in PsA compared with OA (median 0.83 ng/ml, IQR 0.73–3.38 ng/ml; *p* = 6 × 10^−4^) and gout (median 0.97 ng/ml, IQR 0.80–1.48 ng/ml; *p* = 0.004). The same trend was observed for IL-17A, where levels in PsA SF (median 7.77 pg/ml, IQR 3.26–18.14 pg/ml) were higher than both OA SF (median 3.20 pg/ml, IQR 1.55–3.20 pg/ml; *p* = 9.3 × 10^−4^) and gout SF (median 3.20 pg/ml, IQR 2.35–6.40 pg/ml; *p* = 0.027). IFNγ levels were substantially elevated in PsA SF (median 6.44 pg/ml, IQR 3.20–14.35 pg/ml) compared with OA SF (median 3.20 pg/ml, IQR 2.83–4.49 pg/ml; *p* = 0.015). CXCL10, IFNγ, and IL-17A levels between patients with PsA and patients with RA were not significantly different.

### Synovial gene expression is concordant with protein levels

**Fig. 4:**
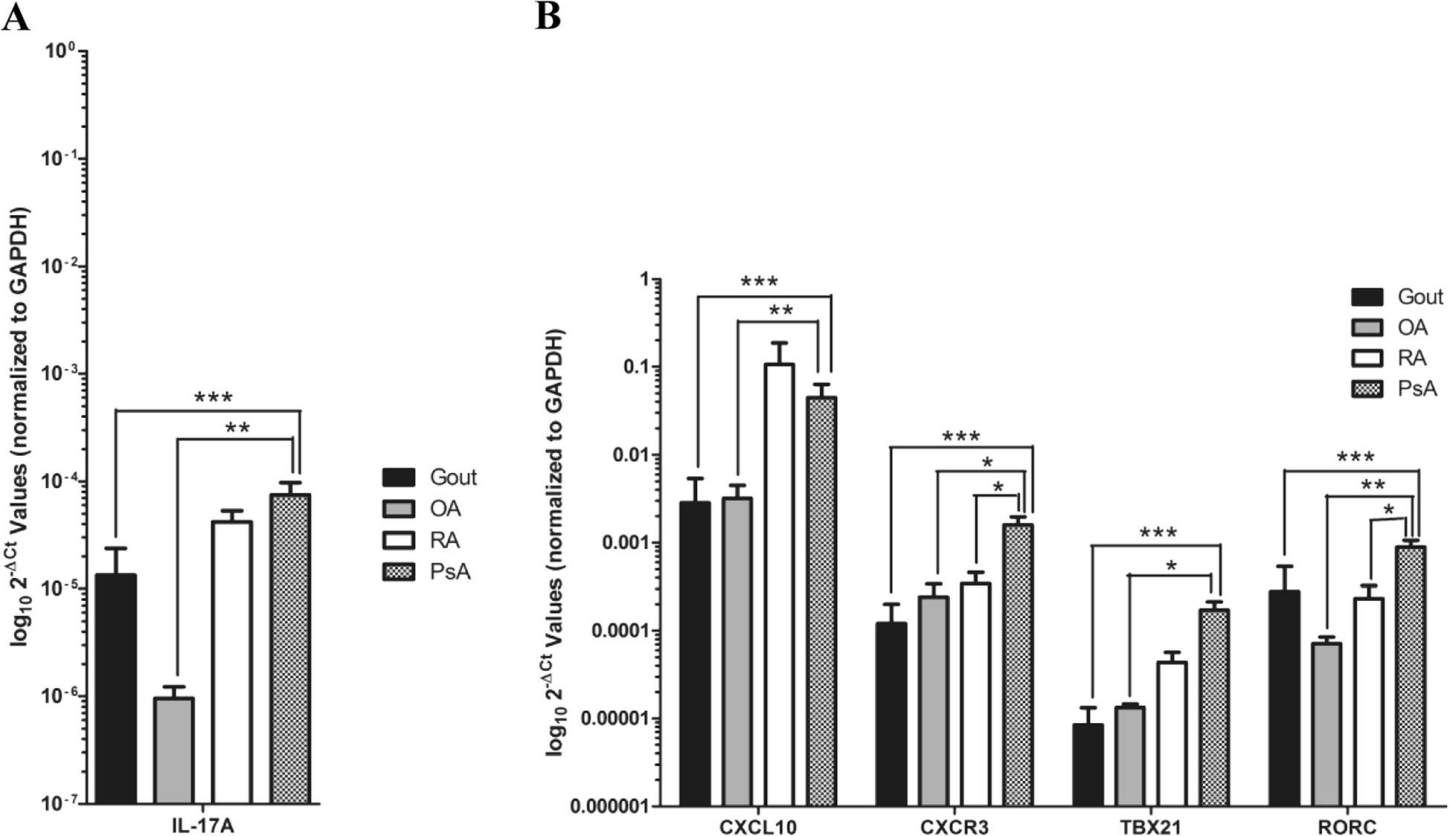
Bar graph depicting gene expression in synovial fluid (SF) cells of patients with psoriatic arthritis (PsA; *n* = 30), osteoarthritis (OA; *n* = 4), gout (*n* = 6), and rheumatoid arthritis (RA; *n* = 5). Error bars indicate SEM. Fold change was calculated using the comparative cycle threshold (ΔΔC_t_) method, and values are shown as log_10_2^−ΔCt^ values to better visualize the difference between groups (see Methods section of main text). **a** Interleukin-17A (*IL-17A*) gene expression was 37.5-fold greater (*p* = 1.5 × 10^−5^) in synovial fluid (SF) of patients with PsA than in those with OA and 19.8-fold greater (*p* = 1.7 × 10^−4^) than in those with gout. **b** Chemokine (C-X-C motif) ligand 10 (*CXCL10*) gene expression was tenfold greater (*p* = 0.007) in SF of patients with PsA than in those with OA and 36.2-fold greater (*p* = 2.6 × 10^−6^) than in those with gout. Chemokine (C-X-C motif) receptor 3 (*CXCR3*) gene expression was 5.3-fold greater (*p* = 0.011) in SF of patients with PsA than in those with OA, 32.3-fold greater (*p* = 1.2 × 10^−6^) than in those with gout, and 3.9-fold greater (*p* = 0.02) than in patients with RA. *TBX21* expression was 5.6-fold greater (*p* = 0.029) in SF of patients with PsA than in those with OA and 25-fold greater (*p* = 0.0001) than in patients with gout. *RORC* gene expression was 8.1-fold greater (*p* = 0.002) in SF of patients with PsA than in those with OA, 19-fold greater (*p* = 0.0001) than in patients with gout, and 3.4-fold greater (*p* = 0.037) than in patients with RA. No difference between patients with PsA and patients with RA was found for *CXCL10*, *TBX21*, and *IL-17A*. Asterisks are used to indicate significant differences between groups, where *p < 0.05, **p < 0.01, and ***p < 0.001. *GAPDH* Glyceraldehyde 3-phosphate dehydrogenase

In a subset of patients from whom sufficient mRNA could be obtained, gene expression was quantified in cells extracted from SF of patients with PsA (*n* = 30) and compared with that of patients with OA (*n* = 4), gout (*n* = 6), and RA (*n* = 5). As shown in Fig. 4, *CXCL10* was tenfold greater (*p* = 0.007) in patients with PsA than in patients with OA and 36.2-fold greater than in patients with gout (*p* = 2.6 × 10^-6^). No significant differences in *CXCL10* levels were found between patients with PsA and patients with RA. There was a significant correlation between CXCL10 mRNA and protein levels in SF (Spearman’s correlation coefficient = 0.428, *p* = 0.02).

A similar finding was obtained for the expression of *CXCR3* in SF cells. *CXCR3* was 5.3-fold greater (*p* = 0.011) in patients with PsA than in patients with OA, 32.3-fold greater (*p* = 1.2 × 10^−6^) than in patients with gout, and 3.9-fold greater (*p* = 0.02) than in patients with RA. IL-17A has previously been shown to play an important role in the pathogenesis of PsA, and anti-IL-17A monoclonal antibodies have recently been approved for clinical use [17, 18]. *IL-17A* gene expression was explored here to gain insight into how the combination of these cytokines/chemokines acts together in PsA. *IL-17A* was found to be 37.5-fold greater (*p* = 1.5 × 10^−5^) in patients with PsA than in those with OA and 19.8-fold greater (*p* = 1.7 × 10^−4^) than in patients with gout. No significant differences between PsA and RA were found for *IL-17A* expression. IFNγ and IL-17A are key effector cytokines of the Th1 and Th17 pathways, respectively. To support our results of elevated IFNγ and IL-17A in PsA SF, we also measured the gene Th1- and Th17-specific transcription factors T-bet (*TBX21*) and RORγt (*RORC*) in SF. *TBX21* was 5.6-fold greater (*p* = 0.029) in patients with PsA than in those with OA and 25-fold greater (*p* = 0.0001) than in patients with gout. No significant differences between PsA and RA were found for *TBX21* expression. *RORC* was 8.1-fold greater (*p* = 0.002) in patients with PsA than in those with OA, 19-fold greater (*p* = 0.0001) than in patients with gout, and 3.4-fold greater than in those with RA (*p* = 0.037).

### *CXCL10* expression is higher in SF than in blood

**Fig. 5:**
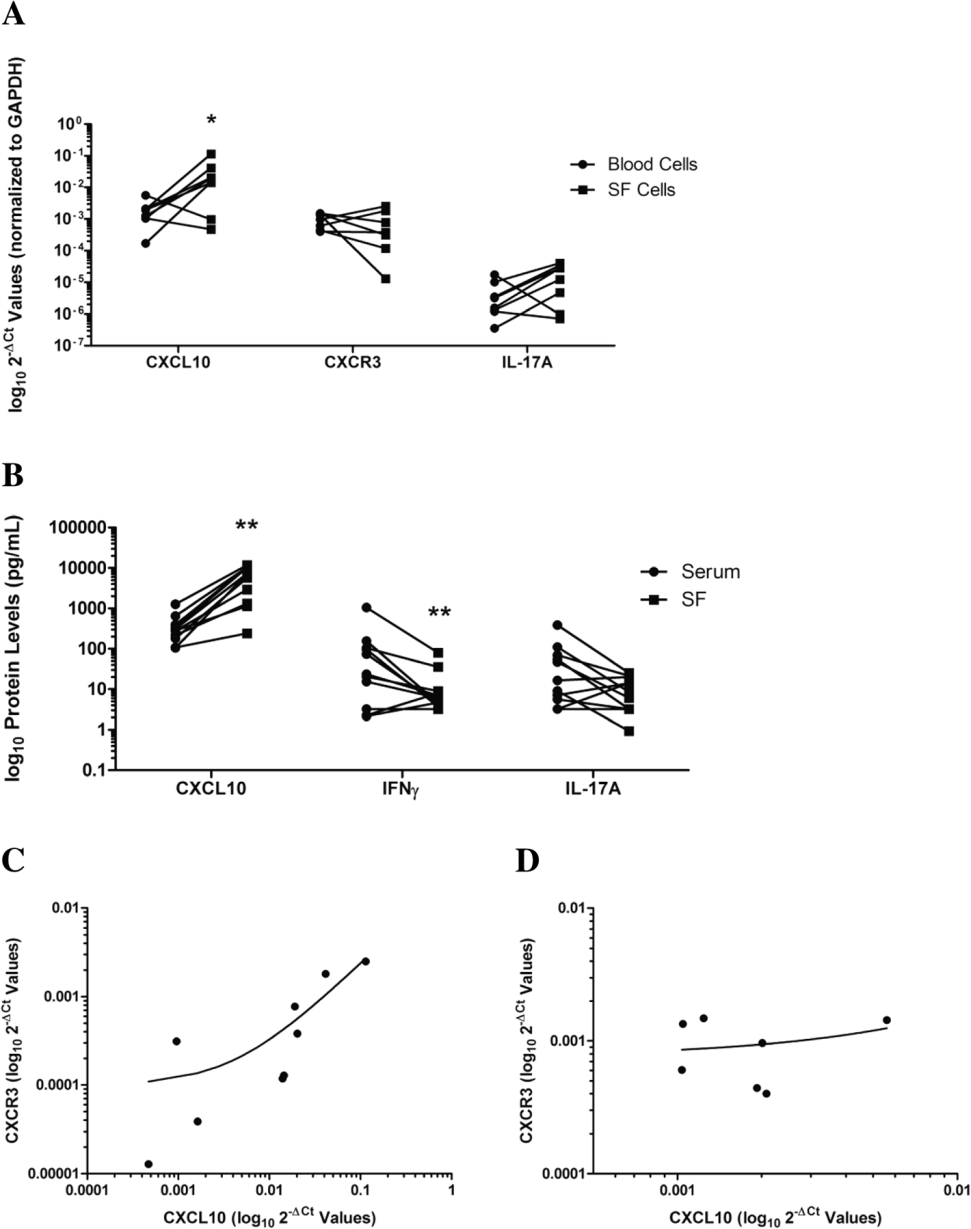
Scatter dot plots of (**a**) paired synovial fluid (SF) and blood gene expression and (**b**) protein levels of cytokine expression in patients with psoriatic arthritis (PsA). Chemokine (C-X-C motif) ligand 10 (*CXCL10*) gene expression was eightfold greater in SF cells (mean ΔC_t_ = 6.55 ± 2.67) than in blood cells (mean ΔC_t_ = 9.55 ± 1.44; *p* = 0.035 by paired Student’s *t* test; *n* = 8). Fold change was calculated using the comparative cycle threshold (ΔΔC_t_) method, and values are shown as log_10_2^−ΔCt^ values to better visualize the difference between groups (see Methods section of text). In paired SF and serum from 11 patients with PsA, CXCL10 levels were significantly elevated in SF, whereas interferon γ (IFNγ) levels were significantly reduced (*p* = 0.001 by Wilcoxon signed-rank test). *CXCL10* and chemokine (C-X-C motif) receptor 3 (*CXCR3*) messenger RNA expression were compared in (**c**) SF and (**d**) blood, and a significant positive correlation was observed in SF (Spearman’s correlation coefficient = 0.883, *p* = 0.0031). Asterisks are used to indicate significant differences between groups, where * = *p*<0.05 and ** = *p*<0.01. *GAPDH* Glyceraldehyde 3-phosphate dehydrogenase, *IL* Interleukin

To compare the localized expression of CXCL10 in the joint with that in the peripheral circulation, we analyzed expression of CXCL10 and other cytokines in paired SF and peripheral blood RNA samples of patients with PsA. In paired SF and blood samples from 11 patients with PsA (Fig. 5a), mRNA expression of *CXCL10* was eightfold greater (*p* = 0.035) in SF cells than in whole blood. No significant difference in gene expression of *IL-17A* and *CXCR3* was observed. As shown in Fig. 5b, CXCL10 protein expression was significantly increased in SF (median 7283.9 pg/ml, IQR 1330–10,362 pg/ml) compared with serum (median 282.06, IQR 180.7–395.8 pg/ml; *p* = 0.001). IFNγ protein levels were significantly reduced (SF median 6.03 pg/ml, IQR 4.47–8.94 pg/ml; versus serum median 23.70 pg/ml, IQR 3.2–104.6 pg/ml; *p* = 0.001), and no significant differences in IL-17A protein levels were observed. We also compared the correlation between *CXCL10* and *CXCR3* mRNA expression in SF (Fig. 5c) and blood (Fig. 5d). There was a significant positive correlation between *CXCL10* and *CXCR3* mRNA levels in SF (Spearman’s correlation coefficient = 0.883, *p* = 0.0031). Interestingly, in some patients (two of eight total), CXLC10 levels were found to be reduced in SF compared with serum. Some observations that may have accounted for this difference included lower total volume of SF (mean volume 9.0 ml, SD 1.4; versus 33.7 ml, SD 26.5), lower concentration of total nucleated cells in SF (0 × 10^6^ cells/L, SD 0 × 10^6^ cells/L; versus 9050 × 10^6^ cells/L, SD 4593 × 10^6^ cells/L), and lower concentration of polymorphonuclear cells in SF (0.25 × 10^6^ cells/L, SD 0 × 10^6^ cells/L; versus 0.70 × 10^6^ cells/L, SD 0.30 × 10^6^ cells/L). This was not reflected in clinical indicators of joint inflammation and differences in blood cell counts, because there were comparable swollen joint counts (2.5, SD 0.71; versus 2.2, SD 2.3), tender joint counts (4.0, SD 4.2; versus 2.2, SD 2.2), blood lymphocytes (1.85 × 10/L, SD 0.07 × 10/L; versus 1.85 × 10/L, SD 0.63 × 10/L), and blood monocytes (0.55 × 10/L, SD 0.07 × 10/L; versus 0.65 × 10/L, SD 0.15 × 10/L) in these patients.

## Discussion

PsA is a chronic inflammatory arthritis that is associated with psoriasis. Within 2 years of the onset of symptoms, many patients (47%) develop erosive disease, and within 10 years, the majority (88%) of the patients develop erosive disease, which leads to dramatic reduction in their overall quality of life and functional capabilities and is associated with an increased mortality rate [19–21]. Disease activity is a predictor for progression of damage; therefore, early treatment will help prevent progressive damage in these patients [22]. Furthermore, initiation of treatment at an early stage of the disease is associated with better outcomes [23, 24]. Soluble biomarkers could serve as an objective measurement tool and could aid in detection of arthritis in patients with psoriasis and in the general population before significant joint damage occurs.

However, the differentiation of PsA from other forms of arthritis at an early stage can be challenging. Clinically, it may be difficult to determine whether pain arising from a joint, tendon, or ligament insertion or from the spine is related to a biomechanical problem, trauma, an autoimmunological inflammatory process, or crystal arthritis [25]. RA and PsA manifest with different symptoms clinically in terms of type and symmetry of joint involvement, joint tenderness, skin and nail involvement, presence of dactylitis and/or enthesitis, and rheumatoid factor presence; therefore, it is easier to distinguish the two conditions by clinical examination. [26]. However, PsA, OA, and gout may not be as easy to distinguish, owing to many similarities in presentation, such as distal interphalangeal joint involvement in both PsA and OA [27]. Although this is due primarily to bony enlargement in OA and joint inflammation in PsA, it is difficult for a nonexpert to distinguish the two. Moreover, some patients with PsA also have OA, making differentiation impossible in some patients [28]. In gout, one can also get joint pain and swelling, as well as redness over the affected joints and swollen digits, which can look like dactylitis, thus making it difficult to clinically distinguish from PsA. In our present study, expression levels of *CXCL10* and *CXCR3* were found to be elevated in SF of patients with PsA as compared with patients with OA and those with gout. *CXCL10* was comparable between patients with PsA and patients with RA, although *CXCR3* was higher in patients with PsA. The same trend as that for *CXCL10* was observed for the Th1-specific transcription factor T-bet (*TBX21*), providing support for increased IFNγ production and CXCL10 within PsA SF. This suggests that CXCL10 may play analogous roles in patients with PsA and patients with RA, but it may distinguish patients with PsA from those with OA and gout.

Elevated levels of CXCL10 in peripheral fluids have previously been described as a marker of host immune response, especially Th1-orientated T cells [29]. CXCL10 was reported to be elevated in several autoimmune diseases, including autoimmune thyroiditis, Graves disease, type 1 diabetes (T1D), systemic lupus erythematosus (SLE), localized scleroderma, and RA [29–32]. CXCL10 is present in dermal infiltrate and keratinocytes of active psoriatic plaques [33, 34] as well as in PsA SF [9]. Proost et al. reported comparable expression of CXCL10 in SF from patients with PsA and patients with RA, consistent with our findings [9]. In serum from patients with PsA and psoriasis, CXCL10 expression has been reported as either elevated [33, 35, 36] or unchanged [37, 38] compared with that of control subjects. Differences in sample sizes and patient characteristics such as disease duration or use of medications may account for these conflicting results. In our cohort, we found elevated CXCL10 in PsA serum compared with that from control subjects, supporting the use of CXCL10 as a biomarker for PsA. Our previous report also suggested that CXCL10 may distinguish patients with PsA from those with PsC [8].

The role of CXCL10 in PsA pathophysiology has not been well defined. Serum CXCL10 levels are negatively correlated to PsA duration [35, 39] and elevated in patients with psoriasis prior to development of PsA, but they drop after PsA onset [8]. Similarly, in T1D, serum CXCL10 levels were high in children and adults, especially at early stages of the disease, but significantly reduced after follow-up. Peripheral blood monocytes and other leukocytes are the main sources of CXCL10 in patients with T1D [40]. The chemokine is also highly expressed in lymphocytes infiltrating into the islet. Upon stimulation with IFNγ and TNFα, β cells release CXCL9, CXCL10, and CXCL11 [41]. These chemokines then promote infiltration of Th1 lymphocytes into the islet through CXCR3, which releases more IFNγ and TNFα, thus perpetuating the positive feedback loop. This in turn leads to the destruction of β cells. In CXCR3-knockout mice, T1D onset is significantly delayed [42]. In active psoriatic plaques, CXCL10 production has been found from keratinocytes and dermal infiltrate, and the migration of NK cells toward CXCL10 has been implicated in psoriasis pathogenesis [33, 43]. In the psoriatic joint, a similar mechanism may occur whereby activated CXCR3-expressing Th1 lymphocytes and/or increased cellular production may be responsible for a concentration of CXCL10 expression. Attenuation of CXCL10 levels is a consequence of therapies currently approved for the treatment of psoriasis and PsA, such as the phosphodiesterase-4 inhibitor apremilast [44] and the TNFα inhibitor etanercept [45, 46], further supporting a role for CXCL10 in the pathogenesis of PsA.

When we compared CXCL10 levels in SF to those in the peripheral circulation, we did observe a dramatic increase in CXCL10 in the majority of patients. In contrast, IFNγ levels were higher in serum, likely reflecting IFNγ produced from multiple sources, including psoriatic plaques [42]. Although CXCR3 levels were not significantly different between SF and peripheral blood cells, there was also a strong positive correlation between *CXCL10* and *CXCR3* mRNA in SF cells, further supporting the notion that localized CXCR3-producing cells may be responsible for the concentration of CXCL10 in the SF. In two patients measured, CXCL10 levels were reduced in SF compared with serum. The lower total SF volume, lower total nucleated cell count in SF, and lower polymorphonuclear cells in SF, as well as equivalent levels of total blood lymphocytes and monocytes, may have contributed to this difference. These observations could have led to reduced inflammatory cells overall, including CXCL10-expressing cells in the SF of these two patients.

Previously, it was thought that PsA is primarily a Th1-mediated disease; however, in recent years, the IL-17/IL-23 axis has been shown to play an important role in disease progression and joint damage. IL-17A is an inflammatory cytokine released from Th17 cells under IL-23 stimulation. The prominent action of IL-17A is attraction of neutrophils to the site of inflammation, generating a powerful immune response [47]. Coculture of healthy human keratinocytes and activated CD4^+^ or CD8^+^ T cells that favor a Th17 phenotype increased production of CXCL10 from keratinocytes [48]. Plasma IL-17 levels are also positively correlated with CXCL10 concentrations in patients with SLE [49]. These studies suggest CXCL10 production may be linked to Th17 cell activation. Indeed, similar to what we saw in CXCL10 and CXCR3 levels, we observed that mRNA expression and protein levels of IL-17A were comparable between PsA and RA samples, in accordance with reports in the literature, and significantly higher than in OA and gout SF samples [50]. We also observed higher mRNA levels of the Th17-specific transcription factor RORγt (*RORC*) in PsA SF than in OA, gout, and RA SF, supporting the involvement of Th17 and a possible link with CXCL10 in PsA. It should be noted that a significant difference in the levels of IL-17A was observed in patients with PsA who also had gene expression measurements completed (median 12.45 pg/ml, IQR 6.21–19.39 pg/ml) as compared with those who did not (3.2 pg/ml, IQR 3.2–7.48 pg/ml; *p* = 0.01). This subgroup of patients with PsA was likely responsible for driving the increased expression of IL-17A in our patients with PsA, possibly owing to an increase in inflammatory cell infiltrate within the SF. Further studies are needed to elucidate the relationship between CXCL10 and the IL-17/IL-23 axis in PsA.

## Conclusions

In conclusion, CXCL10 levels are elevated in PsA serum compared with levels in healthy control subjects, supporting the utility of CXCL10 as a biomarker of PsA susceptibility. In SF, CXCL10 and IL-17A mRNA and protein levels are higher in patients with PsA than in those with OA or gout and similar to those of patients with RA. Furthermore, CXCL10 expression is significantly elevated in the SF compared with the peripheral circulation of patients with PsA. These results indicate that CXCL10 may have an important etiological role in PsA that is analogous to that in RA, and that it may distinguish individuals with PsA from patients with OA and gout.

## Abbreviations

CASPAR: ClASsification for Psoriatic ARthritis criteria
C_t_: Cycle threshold
CXCL10: Chemokine (C-X-C motif) ligand 10
CXCR3: Chemokine (C-X-C motif) receptor 3
DMARD: Disease-modifying antirheumatic drug
GAPDH: Glyceraldehyde 3-phosphate dehydrogenase
IFNγ: Interferon γ
IL: Interleukin
IQR: Interquartile range
mRNA: Messenger RNA
NK: Natural killer
NSAID: Nonsteroidal anti-inflammatory drug
OA: Osteoarthritis
PASI: Psoriasis Area Severity Index
PsA: Psoriatic arthritis
PsC: Psoriasis without psoriatic arthritis
RA: Rheumatoid arthritis
RORγt: RAR-related orphan receptor γt
SF: Synovial fluid
SLE: Systemic lupus erythematosus
T1D: type 1 diabetes
Th: T helper cell
TNFα: Tumor necrosis factor α
